# Nanostructured Conductive Polypyrrole for Antibacterial Components in Flexible Wearable Devices

**DOI:** 10.34133/research.0074

**Published:** 2023-03-10

**Authors:** Yuzheng Wu, Dezhi Xiao, Pei Liu, Qing Liao, Qingdong Ruan, Chao Huang, Liangliang Liu, Dan Li, Xiaolin Zhang, Wei Li, Kaiwei Tang, Zhengwei Wu, Guomin Wang, Huaiyu Wang, Paul K. Chu

**Affiliations:** ^1^ Department of Physics, Department of Materials Science and Engineering and Department of Biomedical Engineering, City University of Hong Kong, Tat Chee Avenue, Kowloon, Hong Kong, China.; ^2^ Center for Human Tissues and Organs Degeneration, Shenzhen Institutes of Advanced Technology, Chinese Academy of Sciences, Shenzhen 518055, China.; ^3^ School of Nuclear Science and Technology, University of Science and Technology of China, Hefei 230026, China.; ^4^ Shanghai Tenth People's Hospital, School of Medicine, Tongji University, Shanghai 200072, China.

## Abstract

The power generated by flexible wearable devices (FWDs) is normally insufficient to eradicate bacteria, and many conventional antibacterial strategies are also not suitable for flexible and wearable applications because of the strict mechanical and electrical requirements. Here, polypyrrole (PPy), a conductive polymer with a high mass density, is used to form a nanostructured surface on FWDs for antibacterial purposes. The conductive films with PPy nanorods (PNRs) are found to sterilize 98.2 ± 1.6% of *Staphylococcus aureus* and 99.6 ± 0.2% of *Escherichia coli* upon mild electrification (1 V). Bacteria killing stems from membrane stress produced by the PNRs and membrane depolarization caused by electrical neutralization. Additionally, the PNR films exhibit excellent biosafety and electrical stability. The results represent pioneering work in fabricating antibacterial components for FWDs by comprehensively taking into consideration the required conductivity, mechanical properties, and biosafety.

## Introduction

Compared to the conventional rigid electronic devices, flexible wearable devices (FWDs) with the desirable flexibility and stretchability are more portable and compatible with human tissues such as skin during body movement [1–7]. Nevertheless, FWDs attached to skin are exposed to the outside environment and are prone to bacteria attachment and colonization, which can lead to skin and respiratory infection [8,9]. Furthermore, bacterial contamination can increase the resistivity of the devices, consequently compromising electrical performance [10]. Although FWDs exhibit some antibacterial effects after electrification, the antibacterial efficiency is normally unacceptable for voltages less than 2.5 V [11]. In general, FWDs should be light and portable, and so, power harvested from a photovoltaic source or sweat is very limited [12–16]. Meanwhile, because FWDs are normally in direct contact with human body, the applied voltage should be restricted to avoid irritating and damaging human tissues [17,18]. Unfortunately, the electricity produced by FWDs is not enough to eradicate bacteria, and better antibacterial strategies are crucial to safe and long-term use of FWDs without bacterial contamination.

Compared to conventional antibacterial materials/devices, construction of antibacterial FWDs is more challenging as both the electrical and mechanical requirements must be satisfied. For example, the components in FWDs should be stable to maintain electrical capacity. Therefore, the ion release strategy is not suitable in spite of the good fundamental understanding, because the structure of the materials may be altered permanently and the device may not function properly after losing ions. In recent years, physical methods including photocatalytic [19], photothermal [20,21], and sonodynamic strategies [22,23] have been proposed and developed to sterilize drug-resistant bacteria [24,25]. Moreover, a non-leaching antibacterial concept has been proposed to preserve the mechanical properties of materials [26]. By preparing special nanostructures such as nanoflakes [26], nanocolumns [27], and nanorods [28], bacteria can be eliminated effectively by mechanical stress or physical puncture of the bacterial membrane [26,28]. However, conventional nanostructures are usually composed of metal oxides that tend to be brittle and have poor electrical conductivity [26,28]. If FWDs are modified with nanostructured metal oxides, then the electrical properties can be compromised and structural collapse of the antibacterial components can occur because of mechanical deformation. To compound the problem, because FWDs are frequently used on skins and sometimes even in contact with wounds, bacterial infection can lead to immune inflammatory response [29]. Therefore, the ideal antibacterial FWDs should be non-leaching, electrically conductive, deformable, and biocompatible.

Polypyrrole (PPy) is a conductive polymer with excellent flexibility and has potential in the design of FWDs [30]. Compared to metals, PPy is light and provides better structural stability in contact with body fluids such as sweat [31]. Compared to other conductive polymers, PPy not only exhibits a large specific capacitance of 480 F/g [32] but also becomes popular for its environmental stability, good conductivity, excellent redox properties, and easy synthesis [33]. Meanwhile, the biosafety of PPy has been verified to avoid excessive immune response [29,34–37]. Although the pristine PPy lacks intrinsic antibacterial ability, nanostructured PPy offers better regulation of bacterial viability. For example, the PPy nanosuckers have been fabricated on carbon textile by galvanostatic electropolymerization and microorganisms are attracted by the PPy nanosuckers to enhance bioelectricity generation in microbial fuel cells [38]. Moreover, PPy can be readily tailored to form different morphologies such as nanowires [39], nanospheres [40], and nanosheets by adjusting the electrolytes or using templates during polymerization [41].

**Fig. 1.**
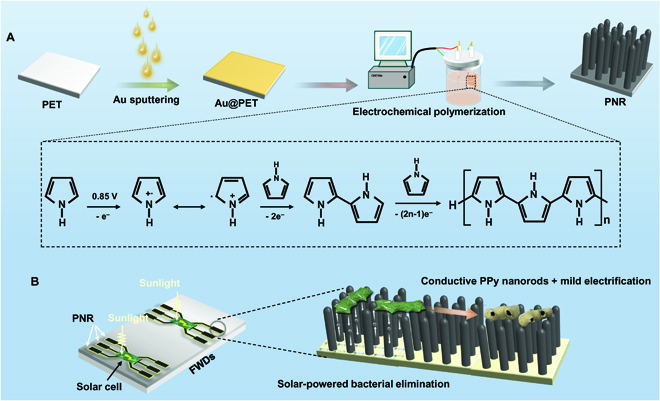
(A) Schematic illustration of the fabrication process of the flexible PNR film and (B) solar-powered elimination of bacteria.

Here, PPy nanorods (PNRs) are prepared by electrochemical polymerization in a high-concentration phosphate-buffered saline (PBS) to produce an antibacterial and biocompatible surface on FWDs. The gold-coated polyethylene terephthalate (Au@PET) film serves as the anode, and the pyrrole monomers are oxidized to form radical cations and interact with the monomer by transferring charges at the applied potential (0.85 V) [42]. The PPy coating then forms a nanorod morphology because of the hydrogen bond interaction in the high-concentration pyrrole/PBS system. Upon contact with bacteria, the PNRs introduce mechanical stress to the bacterial membrane, making the bacteria more sensitive to environmental changes. By means of electrification at a low voltage, the flexible device with PNRs is capable of killing bacteria effectively while retaining satisfactory biocompatibility, electrical conductivity, and mechanical flexibility (Fig. 1).

## Results and Discussion

### Fabrication and characterization of PNR

**Fig. 2.**
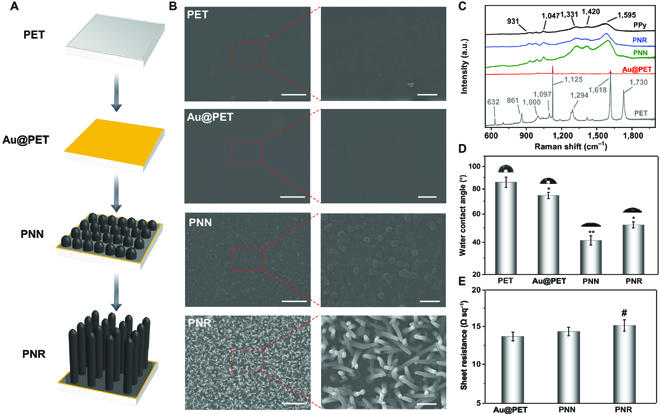
(A) Schematic illustration of the formation of PPy nanorods (PNRs), (B) SEM images, (C) Raman scattering spectra, (D) water contact angles, and (E) sheet resistance of the different samples (scale bars = 5 and 1 μm in the low- and high-magnification SEM images, respectively). *P < 0.05 and **P < 0.01 compared with the PET group; #*P* < 0.05 compared with the Au@PET group. a.u., arbitrary units.

As shown in Fig. 2A and Fig. S1, an Au layer with a thickness of ~150 nm is deposited on the PET substrate to prepare the conductive Au@PET film by ion sputtering, and Au@PET then undergoes electrochemical polymerization for surface modification. In the high-concentration PBS/Py system, nucleation of PPy on Au@PET forms a dense PPy nano-nodular (PNN) layer. During polymerization, the electrostatic interactions and hydrogen bonding between the positively charged Py and HPO_4_^2−^/H_2_PO_4_^−^ restrict the growth direction, resulting in the formation of vertical ordered nanorods [32,43]. The scanning electron microscopy (SEM) images of PET, Au@PET, PNN, and PNR are depicted in Fig. 2B. Compared to the flat PET and Au@PET, nano-nodules emerge from the PNN surface after PPy deposition for 10 min. After polymerization of 30 min, the nano-nodules morph into nanorods with a top diameter of about 170 nm and height of 3 μm (Fig. S2).

The films are analyzed by Raman scattering, and as shown in Fig. 2C, the characteristic Raman peaks from the PET of Au@PET diminish because the Au layer blocks the Raman signal. As for PNN and PNR, the peaks of PPy are observed, confirming successful preparation of the PPy coating (Table S1). These 2 samples show peaks at 931 cm^−1^ (ring deformation associated with dictation), 1,047 cm^−1^ (symmetrical CH in-plane bending and NH in-plane deformation), 1,331 cm^−1^ (antisymmetric in-ring CN stretching), 1,420 cm^−1^ (CC and CN stretching), and 1,595 cm^−1^ (an overlap of CC in-ring and CC inter-ring stretching) [36], which are consistent with the spectra of the PPy film. The x-ray photoelectron spectroscopy (XPS) spectrum of PNR also exhibits a dominant signal from PPy (Fig. S3). As the nanostructured PPy increases the surface roughness, the hydrophilicity is altered and the water contact angles decrease to 41.37 ± 3.06° on PNN and 52.13 ± 2.18° on PNR (Fig. 2D). Although the sheet resistance of PNR increases slightly to 15.17 ± 0.76 Ω sq^−1^ (Fig. 2E), it is still within the usable range for FWDs [15,44,45].

### Antibacterial effects of PNR-modified FWDs

**Fig. 3.**
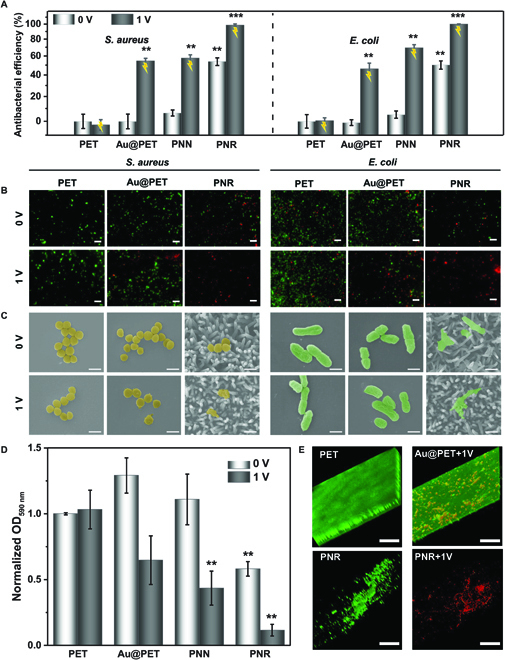
(A) Antibacterial efficiency of the different samples, (B) fluorescence images of bacteria after different treatments and live/dead staining (scale bars = 20 μm), (C) SEM images of the bacteria after different treatments (scale bars = 1 μm) with *S. aureus* shown in gold and *E. coli* in green, (D) quantitative measurement of the antibiofilm performance by crystal violet staining, and (E) 3D morphology of the fluorescent-stained biofilms (scale bars = 25 μm). **P < 0.01 and ***P < 0.001 compared to the PET group.

The antibacterial effects against gram-positive *Staphylococcus aureus* (*S. aureus*) and gram-negative *Escherichia coli* (*E. coli*) on PET, Au@PET, PNN, and PNR are evaluated by counting the bacteria detached from the samples with or without electrification. The PET, Au@PET, PNN, and PET+1V groups exhibit no evident antibacterial activity, but PNR, Au@PET+1V, PNN+1V, and PNR+1V inhibit the growth of bacteria as shown in Fig. S4. As shown in Fig. 3A, only the PNR film is antibacterial in the absence of electrification, showing antibacterial rates of 53.8 ± 4.1% against *S. aureus* and 50.2 ± 4.3% against *E. coli*. After applying 1 V for 3 min, PNR sterilizes 98.2 ± 1.6% of *S. aureus* and 99.6 ± 0.2% of *E. coli*. The antibacterial efficacy is substantially better than that of Au@PET+1V (54.5 ± 2.7% against *S. aureus* and 46.2 ± 5.8% against *E. coli*) or PNN+1V (57.5 ± 3.5% against *S. aureus* and 69.2 ± 3.6% against *E. coli* ).

Live/dead staining is performed to evaluate the viability of *S. aureus* and *E. coli* after the different treatments. The live bacteria emit green fluorescence and dead bacteria emit red fluorescence (Fig. 3B and Fig. S5). After culturing for 3 h, most of the bacteria on PET, Au@PET, and PNN survive as only green fluorescence is detected. On the other hand, red fluorescence representing dead bacteria becomes apparent and almost no green fluorescence is detected from PNR+1V. The SEM images show that both *S. aureus* and *E. coli* on PET, Au@PET, PNN, and PET+1V have the normal shape with intact membranes (Fig. 3C and Fig. S6). In contrast, the bacteria cultured on PNR, Au@PET+1V, and PNN+1V have an irregular shape. In particular, the nanorods on PNR protrude into the bacteria, altering the membrane shape, and the bacteria on PNR+1V are damaged severely with disrupted membranes. These results demonstrate that the nanorod structure endows PPy with excellent antibacterial properties in spite of mild electrification (1 V).

The antibiofilm performance of conductive films is further evaluated with the PET group serving as the control group. *E. coli* growth is significantly reduced on the PNR+1V versus PET group based on the normalized crystal violet absorbance (Fig. 3D). The intensity of crystal violet staining of the Au@PET and PNN groups is slightly higher than that of PET group because the grooves between the nanostructure contain more extracellular matrix produced by the bacteria in the biofilm. Fluorescent staining and crystal violet results show that uniform and dense biofilms are formed on the PET, Au@PET, PNN, and PET+1V groups, whereas the bacteria densities on PNR, Au@PET+1V, and PNN+1V groups are smaller (Fig. 3E and Fig. S7). Specifically, PNR+1V only shows a few numbers of bacteria colonies emitting red fluorescence, which represents dead bacteria. Therefore, the PNR+1V group delivers the best antibiofilm performance, and the better antibiofilm capacity of PNR+1V than other groups with electrification further verifies the importance of the intrinsic bactericidal ability of nanostructured PNR.

### Antibacterial mechanism

**Fig. 4.**
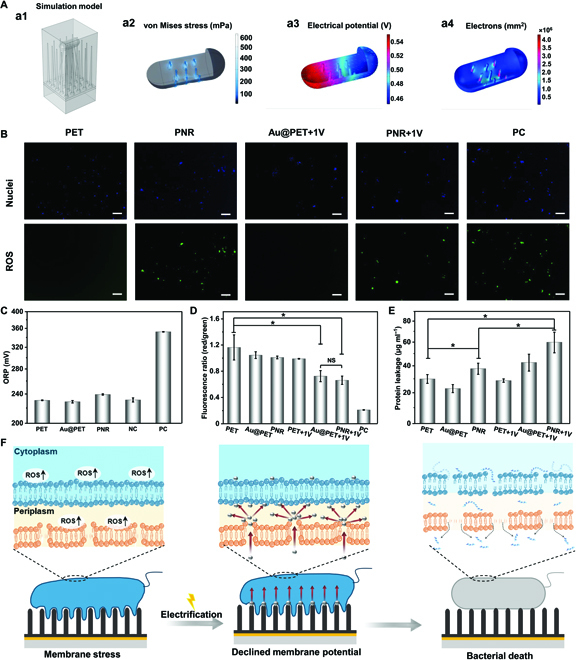
(A) Simulation model and simulated interactions between the bacteria and PNRs, (B) fluorescence images of intracellular ROS for *E. coli* after different treatments (scale bars = 20 μm), (C) ORP values of normal saline incubated with different samples for 3 h, (D) membrane potentials and (E) protein leakage of *E. coli* after different treatments, and (F) schematic illustration of the plausible antibacterial mechanism for PNR+1V. *P < 0.01 and NS represents no significant difference.

According to the experimental observation, the nanorods and electrification contribute to bacterial sterilization. To elucidate the mechanism, simulation is conducted to study the interactions between PNR and *E. coli*. Figure 4A1 shows the simulation model based on the COMSOL software. As bacteria come in contact with the PNRs, no membrane stress is induced by the nanorods without the effects of gravity (Fig. S8A). Under gravity, stress appears and concentrates at the contact site between the nanorods and bacterial membrane because of the weight of *E. coli* (Fig. 4A2 and Fig. S8B), consequently producing membrane deformation in the bacteria (Fig. S8C). The bacterial stress caused by external stimuli gives rise to disorder in the intracellular physiological environment [26,46]. As shown in Fig. 4B and Fig. S9, apparent reactive oxygen species (ROS) signals are detected from the bacteria cultivated on the samples with nanostructured PPy, but other groups exhibit negligible ROS signals (Fig. S10). The oxidation–reduction potential (ORP) value of normal saline containing PNRs is similar to those of other samples (Fig. 4C), demonstrating that no substance is released from the PNR to change the ORP value of the buffer. Therefore, the higher intracellular ROS level of the PNR group is related to the nanorod morphology.

Owing to the good conductivity of Au and PPy, electrification creates an electrical potential in both the Au layer on Au@PET (Fig. S11) and the PPy layer on PNRs (Fig. S12). Consequently, electrons flow through the bacteria and accumulate in some parts of the membrane when a voltage of merely 1 V is applied (Fig. 4A3 and A4). In the physiological environment, a significant decline in the red/green fluorescent ratio signaling decreased membrane potential is observed from the bacteria on the Au@PET+1V and PNR+1V groups compared to the other groups (Fig. 4D and Fig. S13). The smaller membrane potential can be attributed to the multilayer membrane structure with different conductivity. In the inactivated state, the inner membrane shows a resting potential because of different ion concentrations on the medial and lateral sides [47]. The fluorescent membrane potential indicating dye, DiOC_2_(3), aggregates on the inner side of the membrane and emits red fluorescence because of the stable membrane potential. The inner membrane of *E. coli* is surrounded by cytoplasm and periplasm on the medial and lateral sides, respectively [48]. The conductivity of the cell wall (500 mS m^−1^) is higher than that of the cytoplasm (100 mS m^−1^), and hence [49], when electrons flow through the bacteria, the membrane containing periplasm can accommodate more electrons than cytoplasm. Because the outside membrane surface adjacent to periplasm is positively charged [47], introduction of electrons to periplasm neutralizes the charges on the outside of the membrane, thereby dissipating the membrane potential and reducing red fluorescence from the bacteria.

As shown in Fig. 4E, drastic protein leakage is observed from the PNR+1V group, furnishing evidence that the bacterial membrane is destroyed. The antibacterial mechanism of PNR+1V is illustrated in Fig. 4F based on stimulation by membrane stress and electrification. On the PNR surface, the nanorods introduce stress to the bacterial membrane, leading to intracellular physiology disorder. Upon electrification, electrons flow through the nanorods and accumulate in the bacterial periplasm, causing depolarization of the membrane potential. The mechanical stress and depolarized membrane disrupt multiple cellular functions including macromolecular biosynthesis, active transport, and peptidoglycan formation [50–52]. Ultimately, the bacterial envelope subjected to stress and depolarization loses the mechanical integrity and protein leakage and death ensue.

### Practical implementation and biosafety

**Fig. 5.**
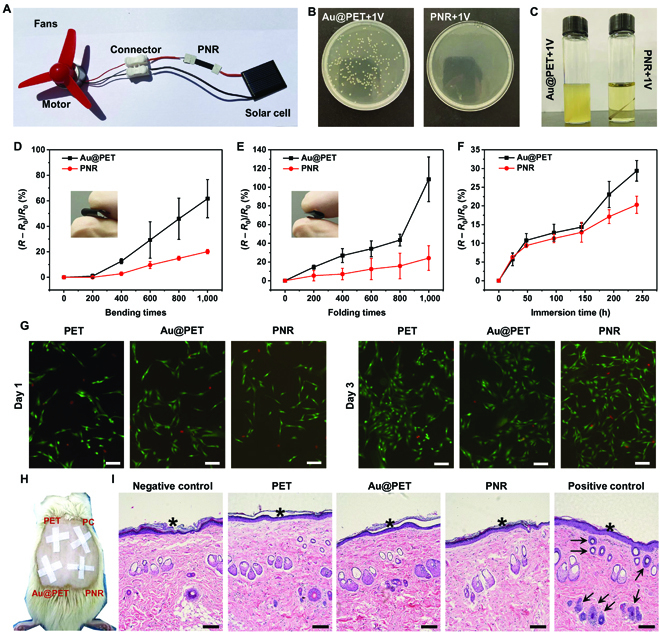
(A) Photograph of the simulated solar-powered bacteria elimination system; (B) colonies of bacteria detached from the different electrified samples; (C) bacteria-contaminated samples after electrification by solar power for 3 min followed by culturing in the LB medium for 12 h; sheet resistance changes of Au@PET and PNR upon (D) bending, (E) folding, and (F) immersing in simulated sweat; (G) live/dead fluorescence images of MRC-5 fibroblasts after culturing on the different samples for 1 and 3 d (scale bars = 100 μm); (H) photograph showing the skin sensitization; and (I) H&E staining images disclosing the accumulation of inflammatory cells in the skin tissues attached to the samples (black arrows indicate inflammatory cell accumulation and * represents the attached site; scale bars = 100 μm).

As a component of FWDs, PNRs must be durable and compatible with human tissues [2]. In our assessment, the PNRs are connected to the closed-circuit system powered by a 1-V solar cell and motor (Fig. 5A). As shown in Movie S1, the fans are driven by the motor under natural sunlight and the electrical current generated by the solar cell can pass through the PNRs to form the complete circuit. The system with bacteria-contaminated samples is exposed to simulated sunlight (25 A, 550 W) for 3 min. Figure 5B and C and Fig. S14 show that PNRs can eradicate bacteria, but Au@PET shows the remaining bacterial residue after the same electrification with simulated sunlight. It is evident that the conductivity of PNRs is adequate to support the circuit and the nanorods enhance the antibacterial properties in the electrification process.

High-quality FWDs should retain the initial conductivity despite repetitive mechanical deformation [53]. The conventional metal- or semiconductor-based FWDs do not adhere well on flexible polymer substrates, resulting in buckling and wrinkles and irreversible damage. Moreover, repeated mechanical deformation can lead to permanent increase in the electrical resistance of FWDs [54]. In this respect, PPy has a flexible C–C backbone with a higher mass density than most conductive polymers, thereby rendering it more adaptable to mechanical stress [30,55]. The PPy layer with nanorods is combined with an Au film to form a sandwiched structure. Beacuse the Young’s modulus of the PPy film (0.3 to 0.6 MPa) is smaller than that of the Au film (41.9 to 55 GPa) [56–58], under mechanical stress, the stiff Au layer buckles/wrinkles, creating cracks, but PPy exhibits larger deformation. After the deformation releases, the fractured Au pieces are able to slide back to the initial position together with the integrated PPy [54]. As the result, the PNR films reduce the conductivity loss after being bent or folded for 1,000 times (Fig. 5D and E). Meanwhile, the PNR films maintain the conductivity in the deformed state (Fig. S15). In acidic simulated hand sweat, the nanostructured PPy layer protects the Au layer from diffusion and retains the protonation [33], thereby exhibiting a smaller resistance increase than that of Au@PET (Fig. 5F).

Because FWDs are frequently used directly on skin or in contact with tissue fluids, potential irritation should be minimized to prevent skin allergy and physiological toxicity. Therefore, the cytocompatibility is evaluated by live/dead staining and 3-(4,5-dimethylthiazol-2-yl)-2,5-diphenyltetrazolium bromide (MTT) assay. As shown in Fig. 5G, few dead cells emitting red fluorescence can be observed from all the samples. The morphology and density of MRC-5 fibroblasts on PNRs are no different from those of PET and Au@PET. The MTT assay reveals that the fibroblasts on PNRs can proliferate, normally corroborating excellent biosafety (Fig. S16).

To evaluate the biosafety in living organisms, the samples are attached to the back skin of rats for 24 h, and the tissue in contact with the samples undergoes hematoxylin-eosin (H&E) staining afterward (Fig. 5H). As shown in Fig. 5I, accumulation of inflammatory cells (black arrows) can only be observed from the positive control group (potassium thioglycolate). In contrast, the tissues of all the sample-attached skins exhibit no difference from the rat skin without any treatment (negative control), thus verifying the good health. Furthermore, the absence of dermatitis further suggests the excellent biocompatibility of PNR and reveals promising application potential of skin-attached FWDs.

FWDs are mainly used for sensing [59], energy storage [60], and actuation [61]. Hence, bacterial contamination occurs easily but must be mitigated or even eliminated. As the electrical power generated by common FWDs is not enough to eliminate bacteria thoroughly, our results reveal a practical antibacterial strategy capable of killing bacteria with mild electrification while preserving the desirable attributes such as electrical conductivity, mechanical robustness, and biosafety.

To further investigate practicality and commercial potential, the nanostructured conductive PPy is introduced to the surface of the PET-based conductive film. The nanorods in the PPy layer produce membrane stress to decrease the bacterial viability without leaching. This is a more stable antibacterial mechanism than ion release [26]. By means of the close circuit, PNR is capable of transporting electrons and producing electron accumulation in the bacterial membrane. Therefore, ascension of the intracellular ROS level and membrane depolarization are detected from PNR+1V, and the dual stress produces synergistic antibacterial effects at a small voltage. In comparison, the PNR is capable of killing a considerable number of bacteria with about one-third of the voltage applied to Au@PET (Fig. S17). In the simulated solar-powered system, the PNRs not only maintain the close circuit but also eradicate bacteria effectively because of the conductive nanostructured PPy layer. In the sandwiched structure, the conductive PNR film retains nearly all of the conductivity despite repeated mechanical deformation and sweat immersion. Furthermore, owing to the insoluble polymeric structure, the PNR film is biocompatible with fibroblasts and skin of rats. This novel platform offers comprehensive advantages including electrical conductivity, mechanical robustness, and biosafety. Because the PNR coating can be fabricated on a conductive surface, our strategy is universal and applicable to a broad range of substrates including metal-coated films and carbon textiles and thus has immense practical and commercial potential.

## Conclusion

Nanostructured PPy is fabricated on the Au-coated PET to produce antibacterial effects on FWDs. Our investigation of the antibacterial mechanism reveals that stress produced by the PNRs and membrane depolarization synergistically destroy the bacterial membrane even at a small voltage. The electrical stability and biosafety of PNRs are verified by durability and cytocompatibility assessment. In addition to the excellent antibacterial properties, our design comprehensively takes into consideration structural integration, mechanical durability, electrical stability, and biosafety. The novel platform has immense potential in FWDs, and our mechanistic study also provides insights into the design of antibacterial FWDs.

## Materials and Methods

### Reagents

PET films with a thickness of 75 μm were purchased from Feixiang Photoelectric Technology (China), and the pyrrole monomers were obtained from Sigma-Aldrich (USA). The PBS solution (0.2 M, pH of 6.8) was prepared by mixing 51% (v/v) of NaH_2_PO_4_ (0.2 M) and 49% (v/v) of Na_2_HPO_4_ (0.2 M). NaH_2_PO_4_ and Na_2_HPO_4_ were purchased from Energy Chemical (China), and lactic acid was obtained from RDH (Germany). NaCl, NaOH, and urea (99.5%) were purchased from International Laboratory (USA).

### Preparation of flexible conductive films

The PET films were ultrasonically washed in deionized water and ethanol sequentially to remove surface contamination. After drying at room temperature, the PET films were placed in the ion sputtering instrument (JFC-1100E, JEOL), and Au was deposited by magnetron sputtering for 5 min (0.8 kV, 10 mA, distance of 5 cm, 0.1 mbar).

The PNRs were prepared on Au@PET by electrochemical polymerization as described previously [32]. Briefly, 30 ml of PBS (0.2 M, pH of 6.8) containing the pyrrole monomer (0.6 M) was prepared and stored at 25 °C. A 3-electrode system was used in polymerization, in which the Au@PET film, platinum rod, and Ag/AgCl electrode were the working, counter, and reference electrodes, respectively. Electrosynthesis was carried out potentiostatically on the CHI760e at 0.85 versus Ag/AgCl for 30 min. After polymerization, the films with PNRs were rinsed with distilled water to remove the pyrrole monomers and dried at room temperature thereafter.

### Characterization

The morphology of the samples and bacteria was examined by SEM (JSM-IT500, JEOL), and the structure was analyzed using the HORIBA Jobin Yvon Raman microprobe equipped with a 633-nm laser. To prepare a positive control for the Raman spectra, a PPy film was prepared using the protocol described previously [36]. Elemental analysis was performed by XPS on the K-Alpha system (Thermo Fisher Scientific, USA) with Al K_α_ radiation referenced to the Ar 2p peak at 242.4 eV. Depth profiles were acquired by XPS using an Ar sputtering rate of 31.6 nm min^−1^. The water contact angles were determined on the Ramé-hart (USA), and the square resistance was measured using a 4-point probe (HPS2523, Helpass Electronics Technologies Inc.).

### Antibacterial assay

*S. aureus* and *E. coli* were used to evaluate the antibacterial activity. The bacterial suspension (100 μl, 10^7^ ml^−1^) was added to the different samples (5 × 25 mm) and cultured at 37 °C for 3 h. Subsequently, the samples were electrified at 1 V for 3 min using a dc meter (IT6123, ITECH). Afterward, the bacteria were detached ultrasonically from the films in PBS for 2 min that the bacterial suspensions were diluted to the proper concentration, spread on agar culture plates, and incubated at 37 °C for 20 h to form colony-forming units (CFU). The antibacterial rate was calculated by the plate counting method using the following formula: Antibacterial rate = (1 − CFU_experimental group_/CFU_control group_) × 100%, where PET served as the control group.

The Live/Dead BacLight Viability Kit (Thermo Fisher, USA) was utilized to evaluate the real-time viability of bacteria after different treatments according to the manufacturer’s instruction. The bacteria after live/dead staining were observed under a fluorescence microscope (Observer Z1, ZEISS, Germany). To examine the morphology, the bacteria were fixed with 2.5% glutaraldehyde for 2 h and dehydrated sequentially in 30%, 50%, 70%, 90%, and 100% (v/v) ethanol for 10 min. The samples with bacteria were then dried at room temperature, sputter-coated with Au, and observed by SEM.

### In vitro antibiofilm assays

*E. coli* was cultured on various samples to form biofilms, and the antibiofilm performance is evaluated. The electrification groups were charged for 3 min every 8 h during bacteria cultivation. The films were seeded with *E. coli* (100 μl, 10^7^ CFU ml^−1^) and incubated at 37 °C for 3 h. The samples with the bacteria were immersed into 2 ml of the lysogeny broth (LB) medium for 48 h to form biofilms, and the culture medium was refreshed every 12 h. Finally, the antibiofilm ability of different samples was evaluated by crystal violet staining and observing the 3-dimensional (3D) morphology. In crystal violet staining, the samples with biofilms were rinsed in PBS, immersed in the 0.1% crystal violet aqueous solution for 25 min, and gently rinsed with deionized water for 5 min. The bound crystal violet was dissolved in 1 ml of ethanol, and the eluate was analyzed on a multimode reader (BioTek, USA) to determine the absorbance at 590 nm [62]. In the 3D morphology observation, the films with biofilms were stained with the LIVE/DEAD BacLight Bacterial Viability Kit (Thermo Fisher, USA) and observed under a confocal microscope (Leica, TCS SPE, Germany).

### Antibacterial mechanism

Gram-positive *E. coli* was utilized to evaluate the antibacterial mechanism. The intracellular ROS levels of *E. coli* were evaluated by 2′,7-dichlorodihydrofluorescein diacetate (DCFH-DA; Beyotime, China) staining, and 4′,6-diamidino-2-phenylindole (DAPI) staining was performed to locate the bacteria position. The fluorescence signals were detected using a fluorescent microscope (Observer Z1, ZEISS, German) at excitation wavelengths of 488 nm (DCFH-DA) and 358 nm (DAPI). The bacteria treated with 10 mM hydrogen peroxide (H_2_O_2_) served as the positive control, and the intensity of the fluorescent ROS was processed by the ImageJ software.

The samples were immersed in 2 ml of normal saline, and the culture media were collected after culturing (without bacteria) at 37 °C for 3 h. The ORP of the solutions was measured using an ORP detector (STORP1, Ohaus, USA), and the normal saline with/without 10 mM H_2_O_2_ served as the positive and negative control, respectively.

The BacLight Bacterial Membrane Potential Kit (Thermo Fisher) was used to determine the bacterial membrane potential according to the manufacturer’s protocol. To monitor the changes in the membrane potential, the red/green fluorescence ratio of the stained bacteria was measured by flow cytometry (BD FACSCalibur, USA) with a laser emitting at 488 nm. Carbonylcyanide m-chlorophenylhydrazone (CCCP), which increased proton permeability and decreased the membrane potential, was added to the DiOC_2_(3)-stained bacteria to form the positive control.

After the antibacterial treatments, the bacteria on the different samples were rinsed with PBS (100 μl), and the eluates were centrifuged at 5,000 rpm for 5 min. The supernatant was collected to measure the protein concentration using the BCA Protein Assay Kit (Beyotime, China) by detecting the absorbance at 562 nm.

### Electrical and mechanical simulation

The electrical and mechanical properties were simulated using the COMSOL 5.4.3a Multi-Physics Finite Element-Based Solver based on the electrical model as shown in Fig. S13A. Briefly, the PET film (2.5 × 2.5 × 1 μm) was put on the bottom and a 0.15-μm-thick Au layer was deposited on PET. The PRNs (~3 μm in height) were placed on the Au film. The bacteria (length of 1.5 μm, radius of 0.3 μm) were half-embedded into the top of the nanorods to simulate the bacterial status, and normal saline (2.5 × 2.5 × 4 μm) was considered to simulate the moist environment of bacteria. The electrical parameters are listed in Table, and a voltage of 1 V was applied to the left sidewall of the Au film with the right sidewall grounded. The mechanical simulation was carried out using the solid mechanics module, in which a force of 9.8 × 10^−15^ N derived from the bacterial weight was applied to the interface between the nanorods and bacteria. The Young’s modulus was 200 Pa and the Poisson’s ratio was 0.3 in the simulation model [63].

**Table.** Simulation parameter of the materials and bacteria.

Parameters	PET	Gold film	PPy	Bacteria	Normal saline
Conductivity (S m^−1^)	10^−14^	4.52 × 10^7^	5,000	80	80
Relative permittivity	4	0	4.5	0.8	0.8

### Solar-powered bacteria elimination

The *E. coli* suspension (20 μl, 10^7^ ml^−1^) was added onto the conductive films (5 × 25 mm) and cultured at 37 °C for 3 h. They were connected to a motor with fans and a solar cell (1 V; Taobao Marketplace) and placed on the stage of the solar simulator (ABET Technologies). After light exposure for 3 min (25 A, 550 W), the samples were disconnected to evaluate the efficiency solar-driven bacteria elimination. The samples were immersed in the LB medium in a shaker (37 °C, 200 rpm) for 10 h to determine the turbidity of the LB medium. In addition, the bacteria were detached from the samples to determine the antibacterial efficacy by the plate counting method. The connected system was also placed in open space outside the Plasma Laboratory in the City University of Hong Kong, and the fan rotation was monitored using a digital camera.

### Durability test

The samples with dimensions of 13 × 13 mm were prepared to evaluate the durability after mechanical deformation. The samples were bent or folded in half from the middle for 1,000 times, after which the sheet resistance was recorded by 4-point probe. Moreover, the conductivity of the deformed films was determined according to the following relationship: Conductivity = 1/(*R*_sheet_*t), where *t* is the thickness of the conductive coating. The artificial sweat was prepared by dissolving 0.5% NaCl, 0.5% lactic acid, and 0.1% urea in deionized water, and the pH was adjusted to 5.0 with 0.1 M NaOH [64,65]. The Au@PET and PNR films (5 × 25 mm) were immersed in 4 ml of the artificial sweat and incubated at 37 °C. The sheet resistance was monitored for 240 h.

### Viability of fibroblasts

The MRC-3 fibroblasts were cultured in the Dulbecco’s modified Eagle’s medium (Gibco) containing 10% fetal bovine serum (Gibco) and 1% penicillin/streptomycin (Invitrogen) at 37 °C in a 5% CO_2_ atmosphere. In the viability assay, 1 ml of the culture medium containing 2 × 10^4^ of MRC-3 was seeded on the samples (10 × 10 mm) in a 24-well plate. After culturing for 1 and 3 d, the cell viability was evaluated by the MTT assay and SYTO9/PI (Thermo Fisher) staining.

### Skin sensitization test

The Sprague Dawley rats (8 weeks old, male) were provided by the Beijing Vital River Laboratory Animal Technology Company Limited, and the animal experiments and procedures were approved by the Ethics Committee for Animal Research, Shenzhen Institutes of Advanced Technology, Chinese Academy of Sciences (approval number: SIAT-IACUC-210223-YYS-WHY-A1618). During surgery, the hair on the back skin of rat was shaved from a 6-cm × 8-cm area, and the rat was anesthetized by pentobarbital sodium (2%, 2.3 mg kg^−1^ of body weight) via intraperitoneal injection. The sample (1.0 cm × 0.5 cm) was attached and fixed on the exposed back skin with the potassium thioglycolate ointment (Nair, USA), serving as the positive control. After attachment for 24 h, the rats were sacrificed, and the skin tissues around the samples were subjected to H&E staining to evaluate the dermatitis state of the attached sites. The healthy rat skin tissue without any treatment was considered the negative control group.

### Statistical analysis

The scale bars in the SEM and fluorescent images were added to reflect the actual scales. All the quantitative data were expressed as average value ± standard deviation with *n* ≥ 3, and the Student *t* test function in the Excel software was employed to calculate the degree of significant difference. A difference of *P* < 0.05 (*) was considered to be statistically significant and that of *P* < 0.01 (**) or *P* < 0.001 (***) was considered to be highly significant.

## Data Availability

The data are available from the authors upon a reasonable request.

## Supplementary Materials

Supplementary 1 Fig. S1. Depth profiles of Au, C, and O of Au@PET by XPS. Fig. S2. SEM image of the cross sections of PNR. Fig. S3. XPS survey spectrum of PNR. Fig. S4. Colonies of *S. aureus* and *E. coli* detached from different samples. Fig. S5. Live/dead fluorescence images of bacteria on PNN with/without electrification. Fig. S6. SEM images of bacteria on PNN with/without electrification. Fig. S7. 3D morphology of the fluorescently stained biofilms in PET, Au@PET, PNN, and PNN+1V groups. Fig. S8. Membrane status of bacteria in contact with PNRs. Fig. S9. Fluorescence images of intracellular ROS for *E.* co*li* on PNN after different treatments. Fig. S10. Quantitative analysis of ROS staining of *E. coli*. Fig. S11. Simulation of the Au film mode: (A) model, (B) electrical potential distribution, and (C) electric field distribution on the Au film. Fig. S12. Simulation of the PNR mode: (A) model, (B) electrical potential distribution, and (C) electric field distribution around the Au film and nanorod surface. Fig. S13. *E. coli* emitting red/green fluorescence after staining with the bacterial membrane potential kit. Fig. S14. Growth curves of bacteria cultured on Au@PET and PNR after electrifying with solar power for 3 min. Fig. S15. Conductivity changes of PNR under bending status. Fig. S16. MTT assay of MRC-5 fibroblasts after culturing with different samples for 1 and 3 d. Fig. S17. Antibacterial efficiency of Au-deposited PET films against *S. aureus* and *E. coli* at different applied voltages. Table S1. Characteristic Raman peaks of PET and PNR.

Supplementary 2 Movie S1. The working solar-powered circuit.
