# Supplementary material for: Nanostructured Conductive Polypyrrole for Antibacterial Components in Flexible Wearable Devices
Source: Research (Wash D C). 2023 Mar 10;6:0074. doi: 10.34133/research.0074 (PMC10013960; doi:10.34133/research.0074)
Supplement: Supplementary 1 — Fig. S1. Depth profiles of Au, C, and O of Au@PET by XPS. Fig. S2. SEM image of the cross sections of PNR. Fig. S3. XPS survey spectrum of PNR. Fig. S4. Colonies of S. aureus and E. coli detached from different samples. Fig. S5. Live/dead fluorescence images of bacteria on PNN with/without electrification. Fig. S6. SEM images of bacteria on PNN with/without electrification. Fig. S7. 3D morphology of the fluorescently stained biofilms in PET, Au@PET, PNN, and PNN+1V groups. Fig. S8. Membrane status of bacteria in contact with PNRs. Fig. S9. Fluorescence images of intracellular ROS for E. coli on PNN after different treatments. Fig. S10. Quantitative analysis of ROS staining of E. coli. Fig. S11. Simulation of the Au film mode: (A) model, (B) electrical potential distribution, and (C) electric field distribution on the Au film. Fig. S12. Simulation of the PNR mode: (A) model, (B) electrical potential distribution, and (C) electric field distribution around the Au film and nanorod surface. Fig. S13. E. coli emitting red/green fluorescence after staining with the bacterial membrane potential kit. Fig. S14. Growth curves of bacteria cultured on Au@PET and PNR after electrifying with solar power for 3 min. Fig. S15. Conductivity changes of PNR under bending status. Fig. S16. MTT assay of MRC-5 fibroblasts after culturing with different samples for 1 and 3 d. Fig. S17. Antibacterial efficiency of Au-deposited PET films against S. aureus and E. coli at different applied voltages. Table S1. Characteristic Raman peaks of PET and PNR. [file research.0074.f1.docx]

Supplementary Materials

Nanostructured Conductive Polypyrrole for Antibacterial Components in Flexible Wearable Devices

Yuzheng Wu^1^†, Dezhi Xiao^1^†, Pei Liu^1^, Qing Liao^2^, Qingdong Ruan^1^, Chao Huang^1^, Liangliang Liu^1^, Dan Li^1^, Xiaolin Zhang^1^, Wei Li^2^, Kaiwei Tang^1^, Zhengwei Wu^3^*, Guomin Wang^1^*, Huaiyu Wang^2^*, Paul K. Chu^1^*

*1* Department of Physics, Department of Materials Science and Engineering and Department of Biomedical Engineering, City University of Hong Kong, Tat Chee Avenue, Kowloon, Hong Kong, China

*2* Center for Human Tissues and Organs Degeneration, Shenzhen Institutes of Advanced Technology, Chinese Academy of Sciences, Shenzhen 518055, China

*3* School of Nuclear Science and Technology, University of Science and Technology of China, Hefei, 230026, China

Correspondence should be addressed to Zhengwei Wu, [wuzw@ustc.edu.cn](mailto:wuzw@ustc.edu.cn); Guomin Wang, [guomiwang2-c@my.cityu.edu.hk](mailto:guomiwang2-c@my.cityu.edu.hk); Huaiyu Wang, [hy.wang1@siat.ac.cn](mailto:hy.wang1@siat.ac.cn); and Paul K. Chu, [paul.chu@cityu.edu.hk](mailto:paul.chu@cityu.edu.hk)

† These authors contributed equally to this work.

**
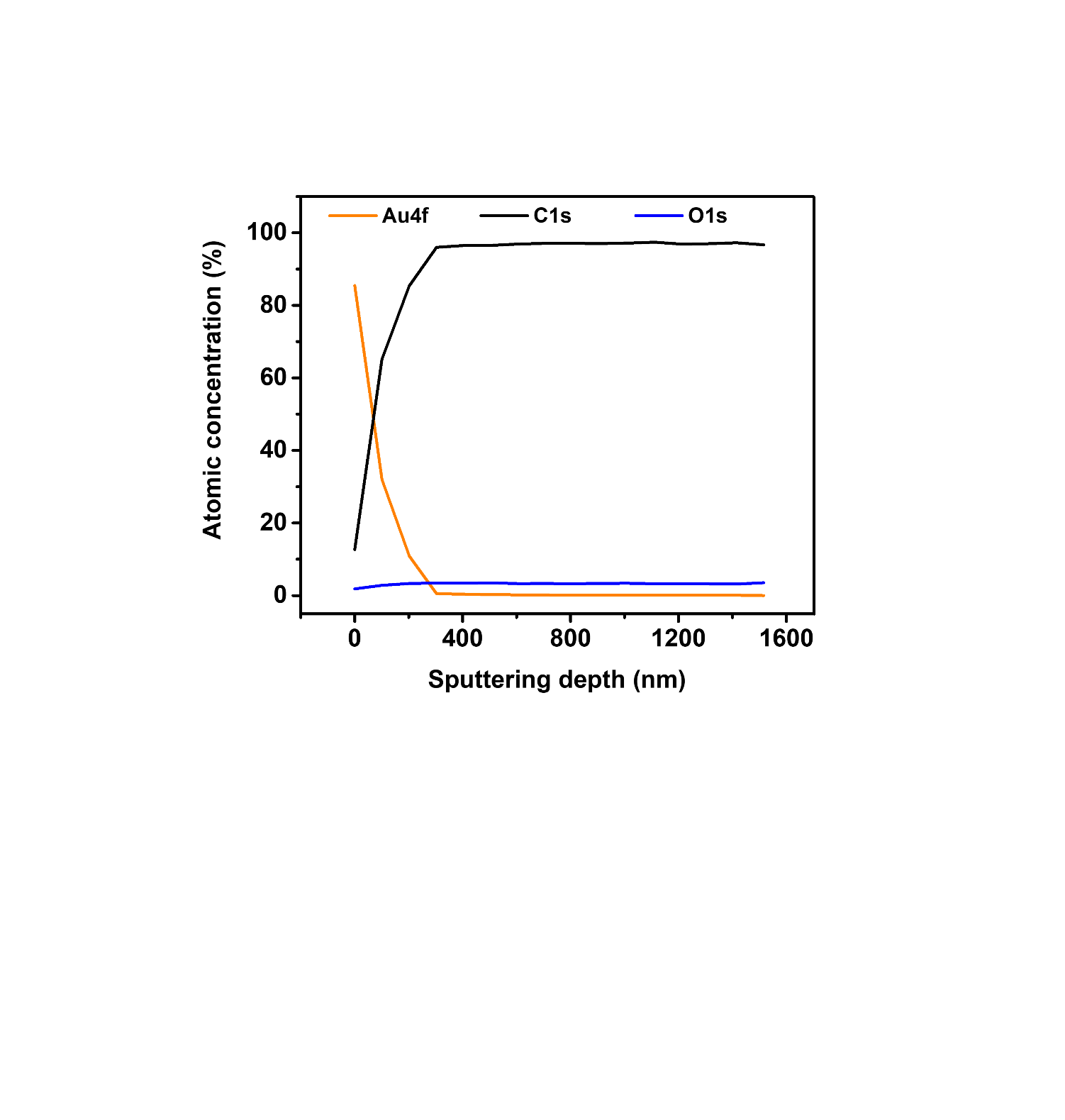
**

**Figure S1.** Depth profiles of Au, C, and O of Au@PET by XPS.

**
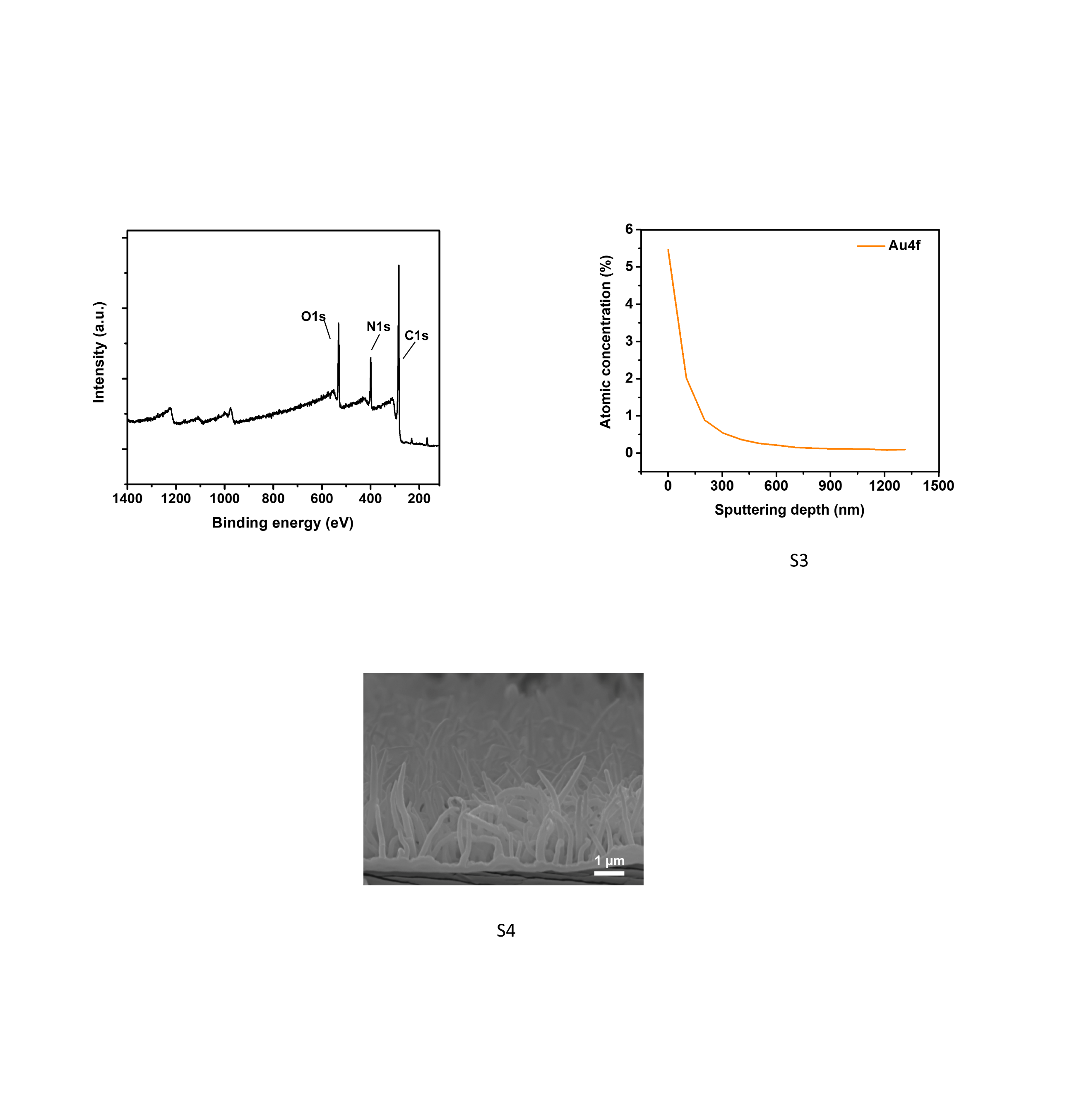
**

**Figure S2.** SEM image of the cross sections of PNR.

**
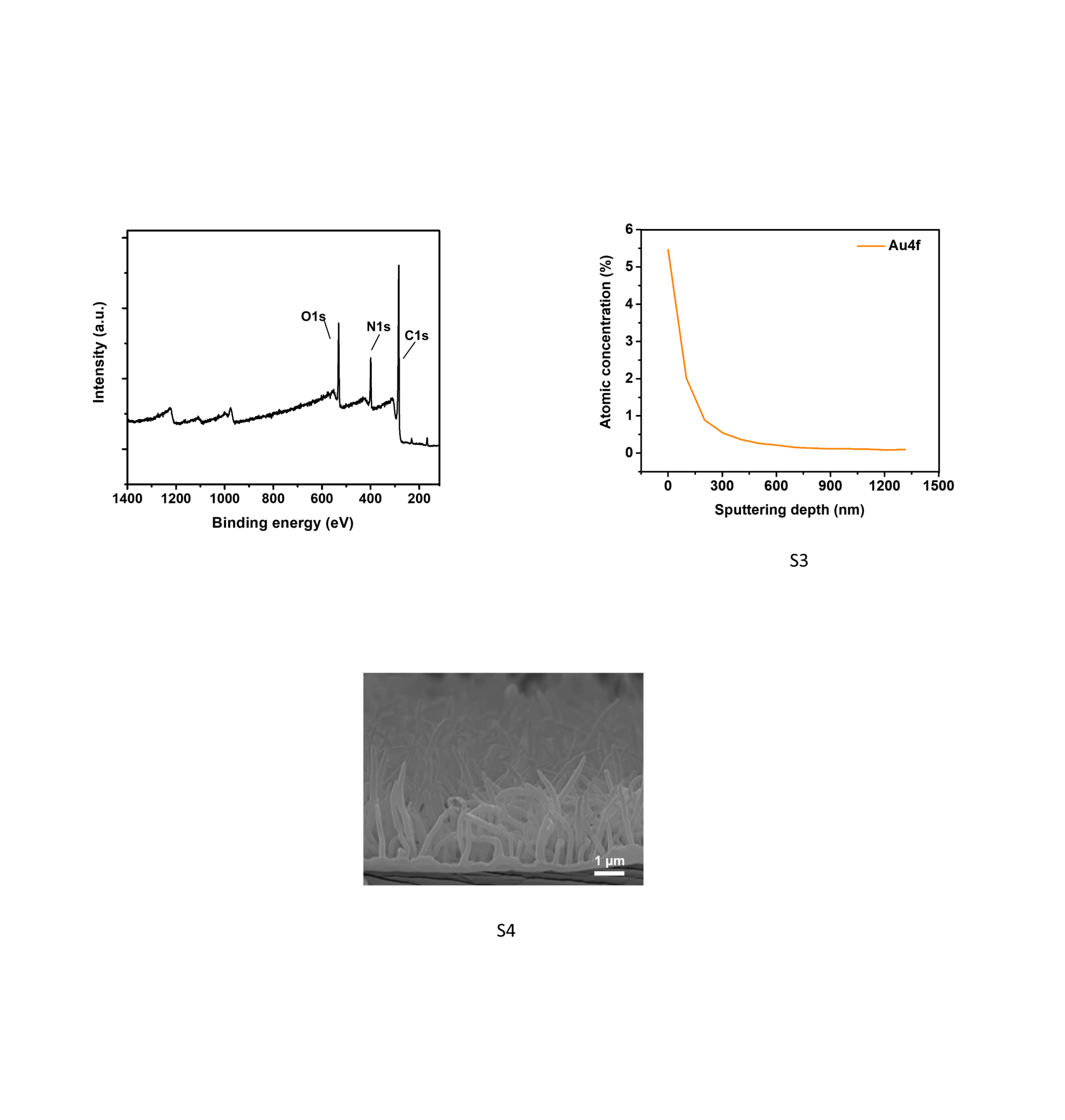
**

**Figure S3.** XPS survey spectrum of PNR.

**
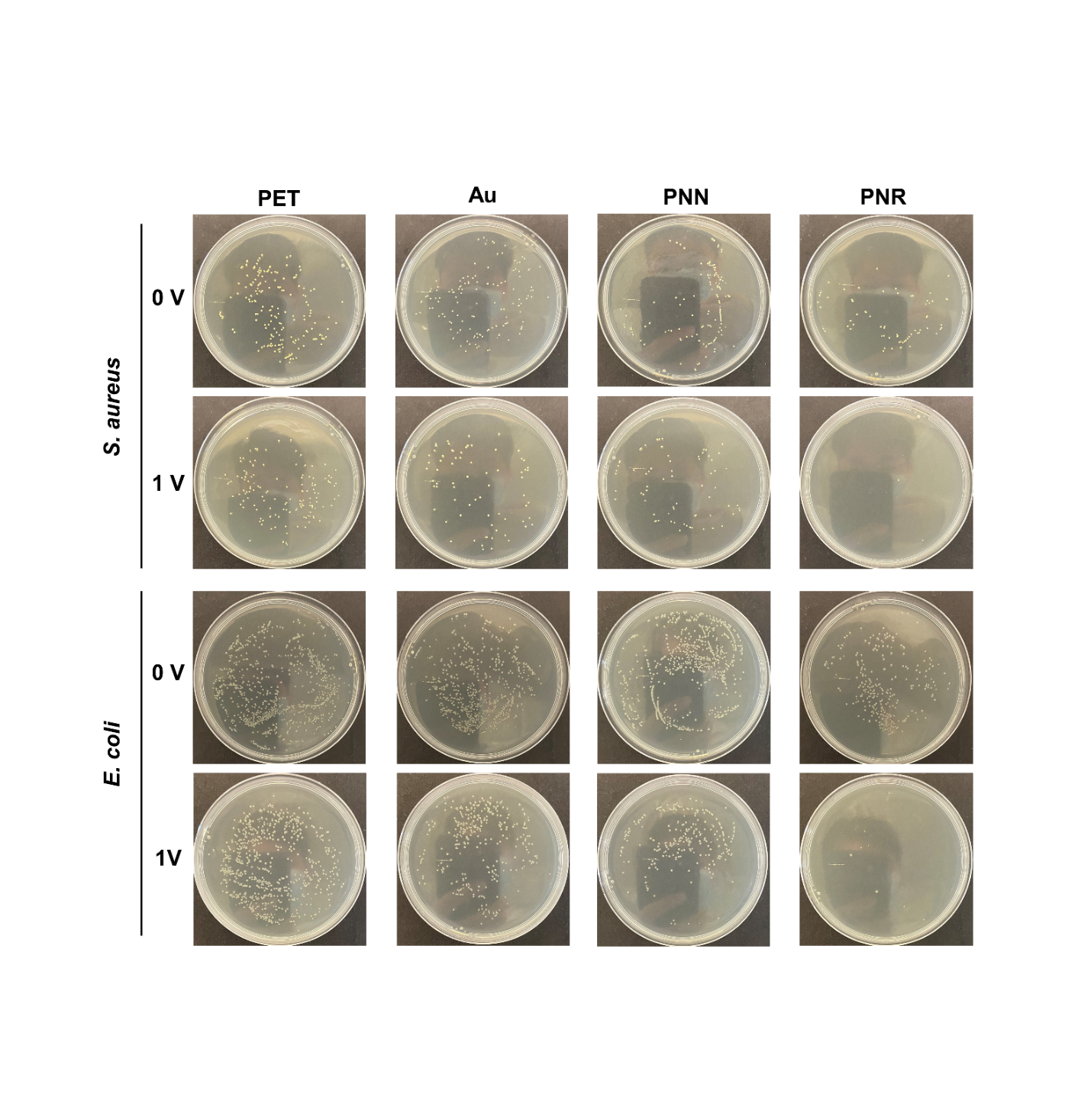
**

**Figure S4.** Colonies of *S. aureus* and *E. coli* detached from different samples.

**
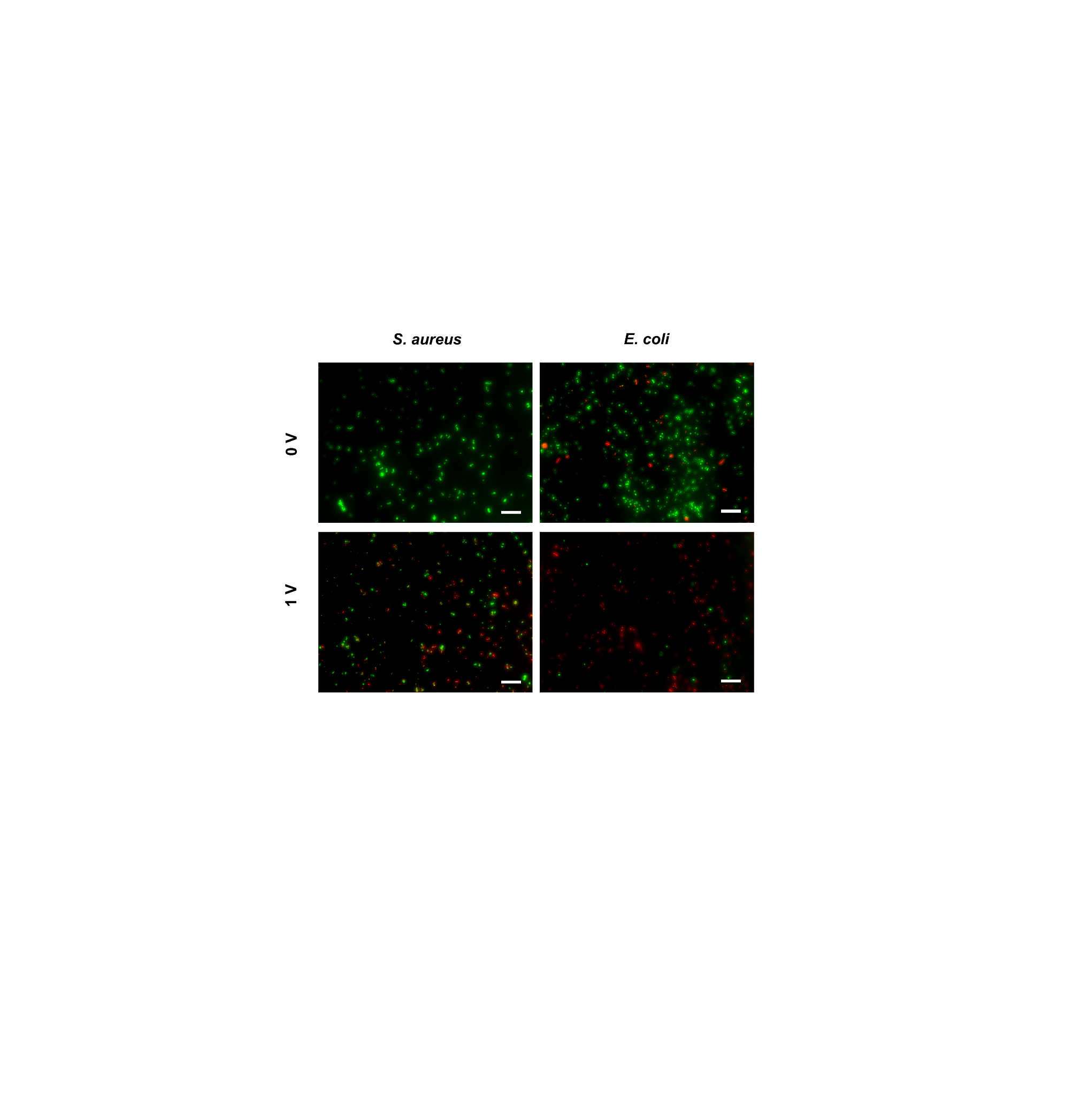
**

**Figure S5.** Live/dead fluorescence images of bacteria on PNN with/without electrification (scale bar = 20 μm).

**
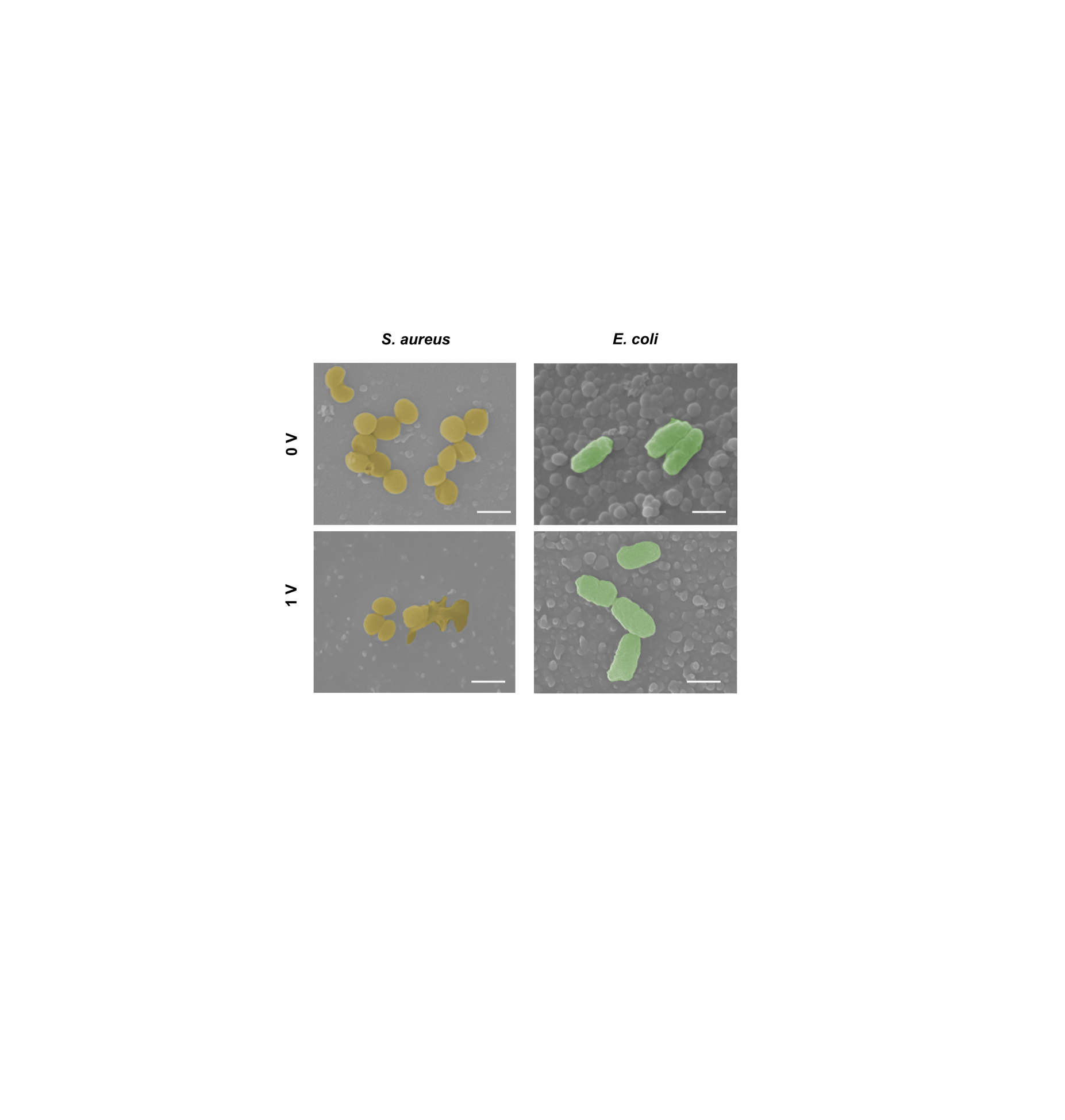
**

**Figure S6.** SEM images of bacteria on PNN with/without electrification (scale bar = 1 μm): *S. aureus* shown in gold and *E. coli* green.

**
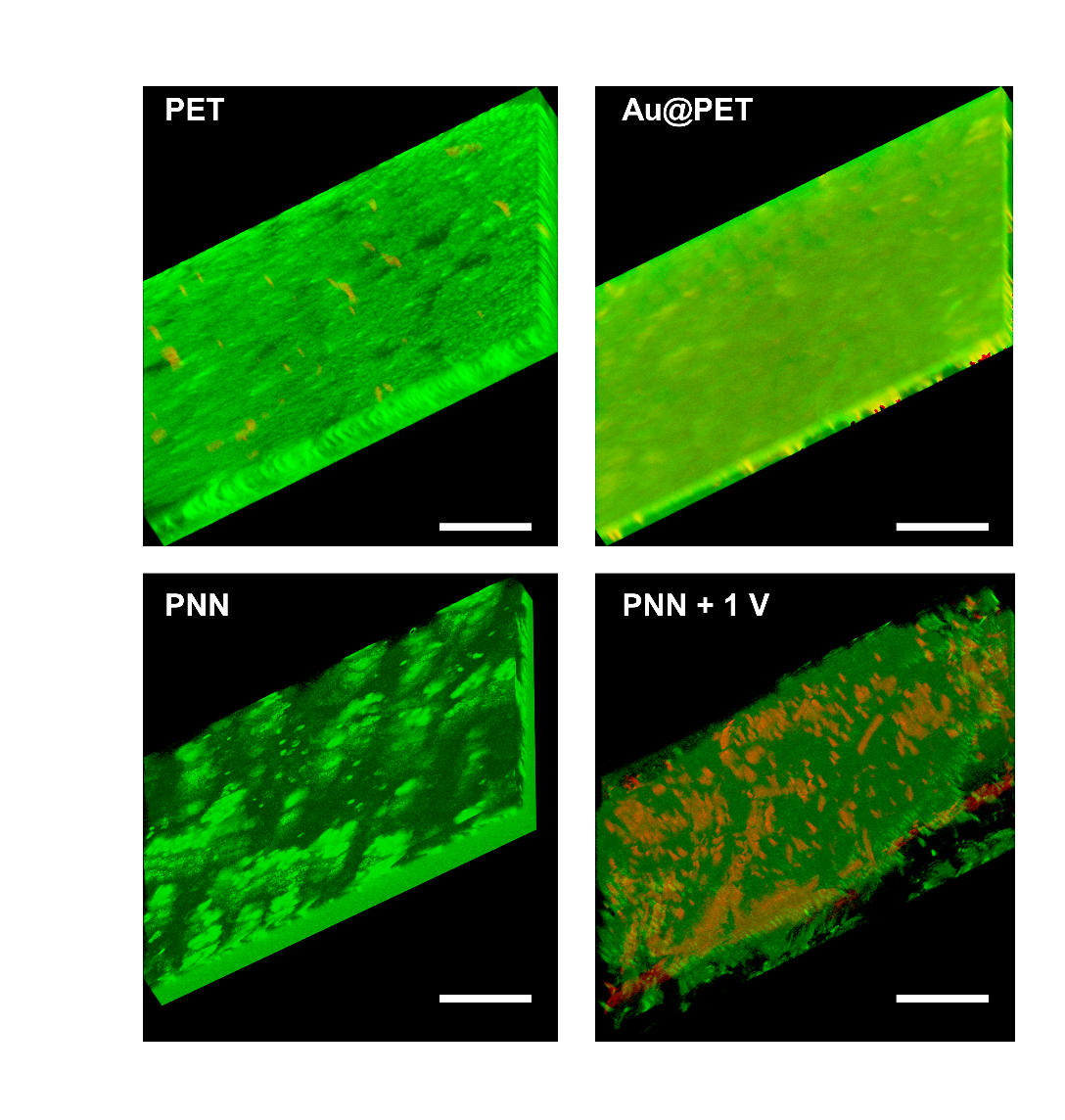
**

**Figure S7.** 3D morphology of the fluorescently stained biofilms in PET, Au@PET, PNN, and PNN + 1V groups (scale bar = 25 μm).

**
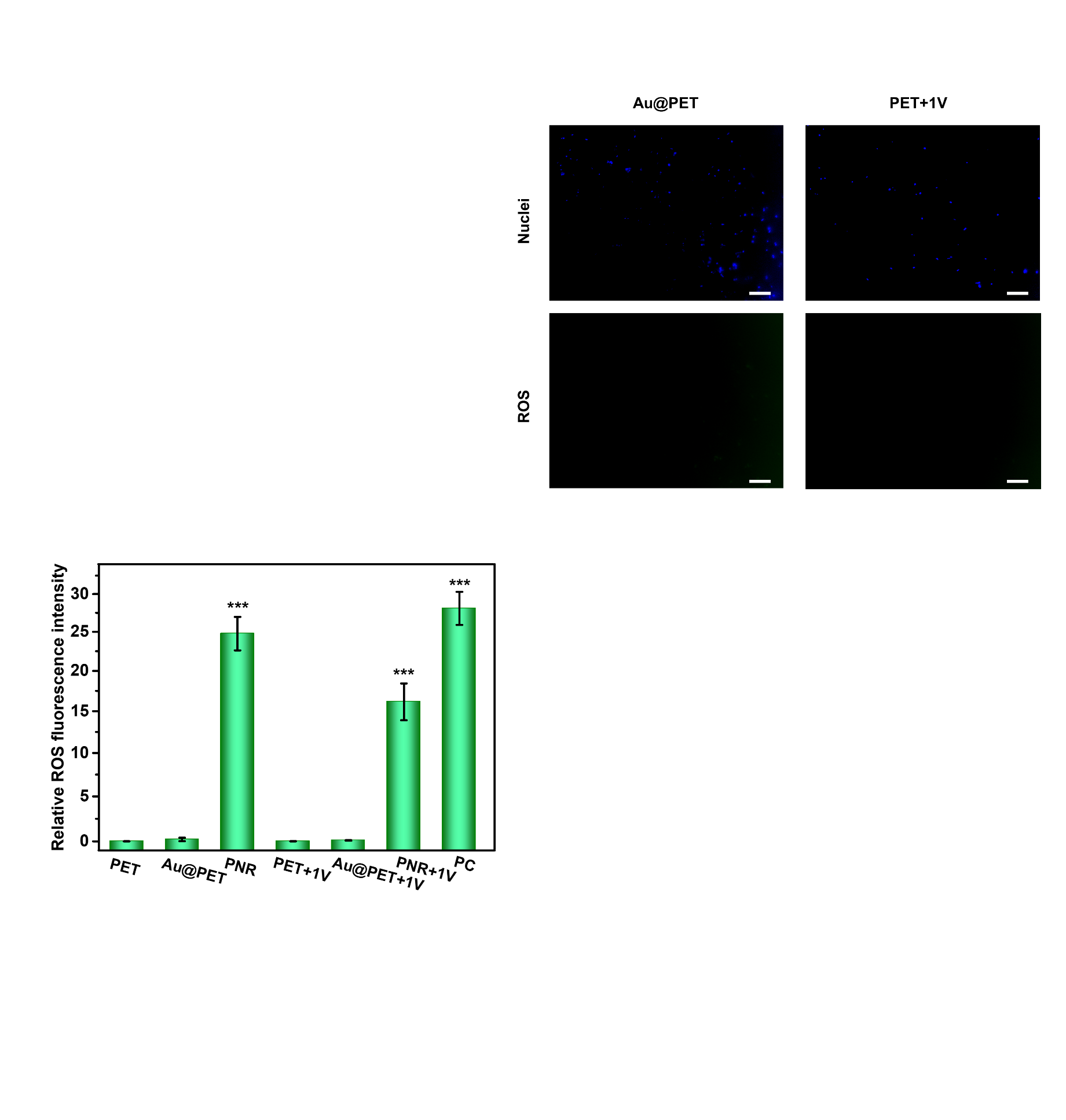
**

**Figure S8.** Fluorescence images of intracellular ROS for *E. coli* on PNN after different treatments (Scale bar = 20 µm).

**
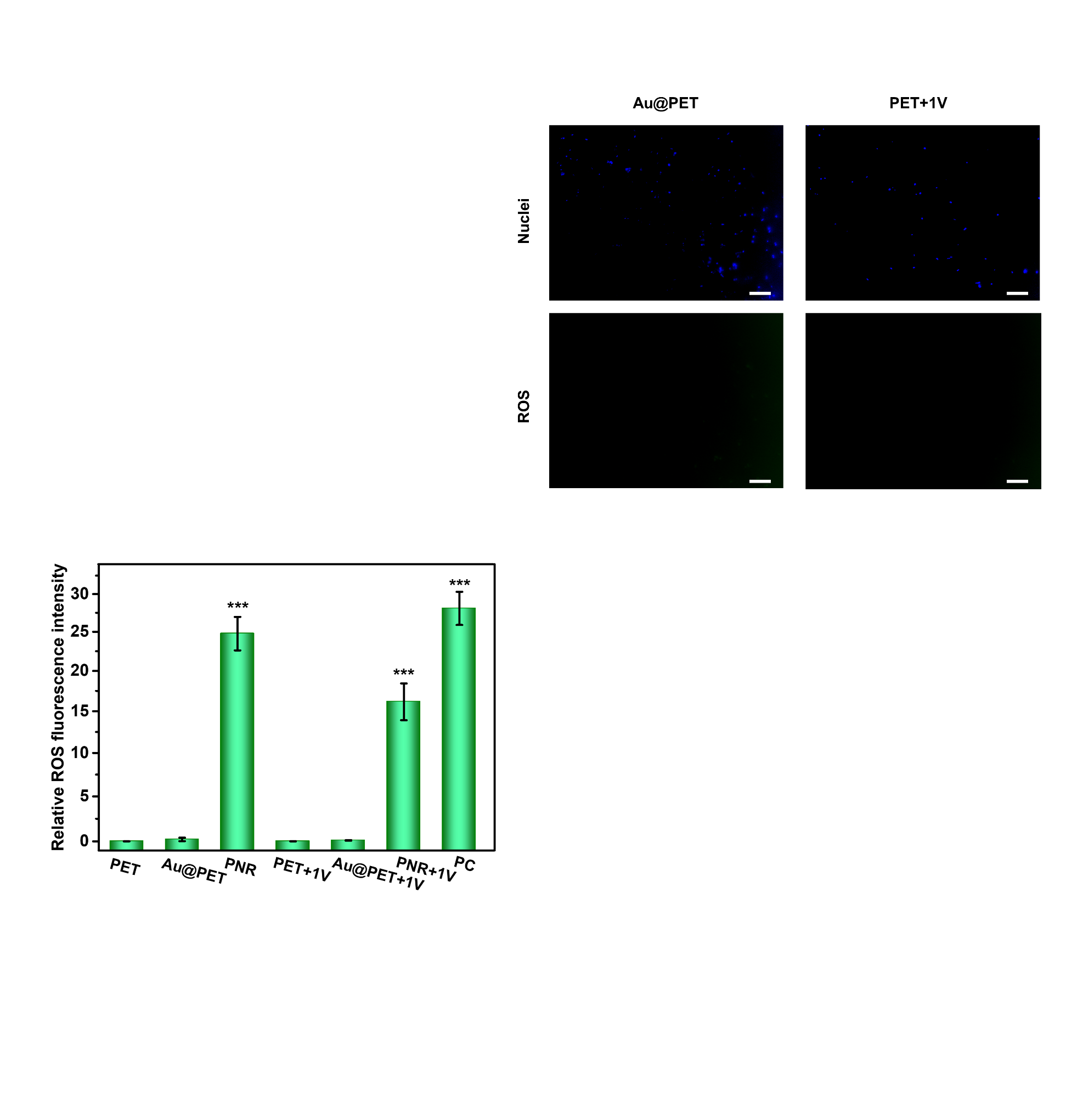
**

**Figure S9.** Quantitative analysis of ROS staining of *E. coli* using the ImageJ software (*** denotes *p* < 0.001 compared to the PET group).

**
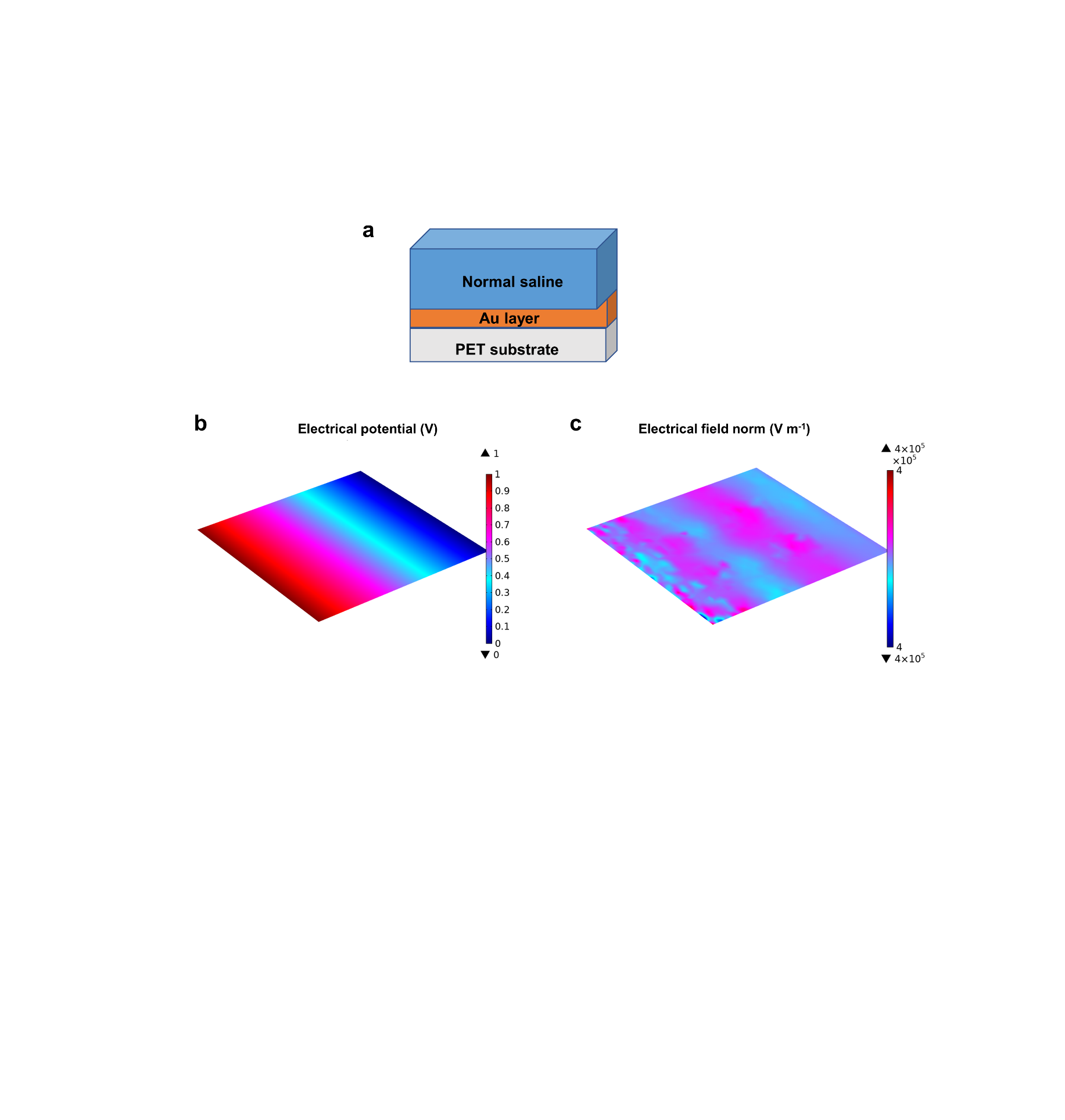
**

**Figure S10.** Simulation of the Au film mode: (a) Model, (b) Electrical potential distribution, and (c) Electric field distribution on the Au film.

**
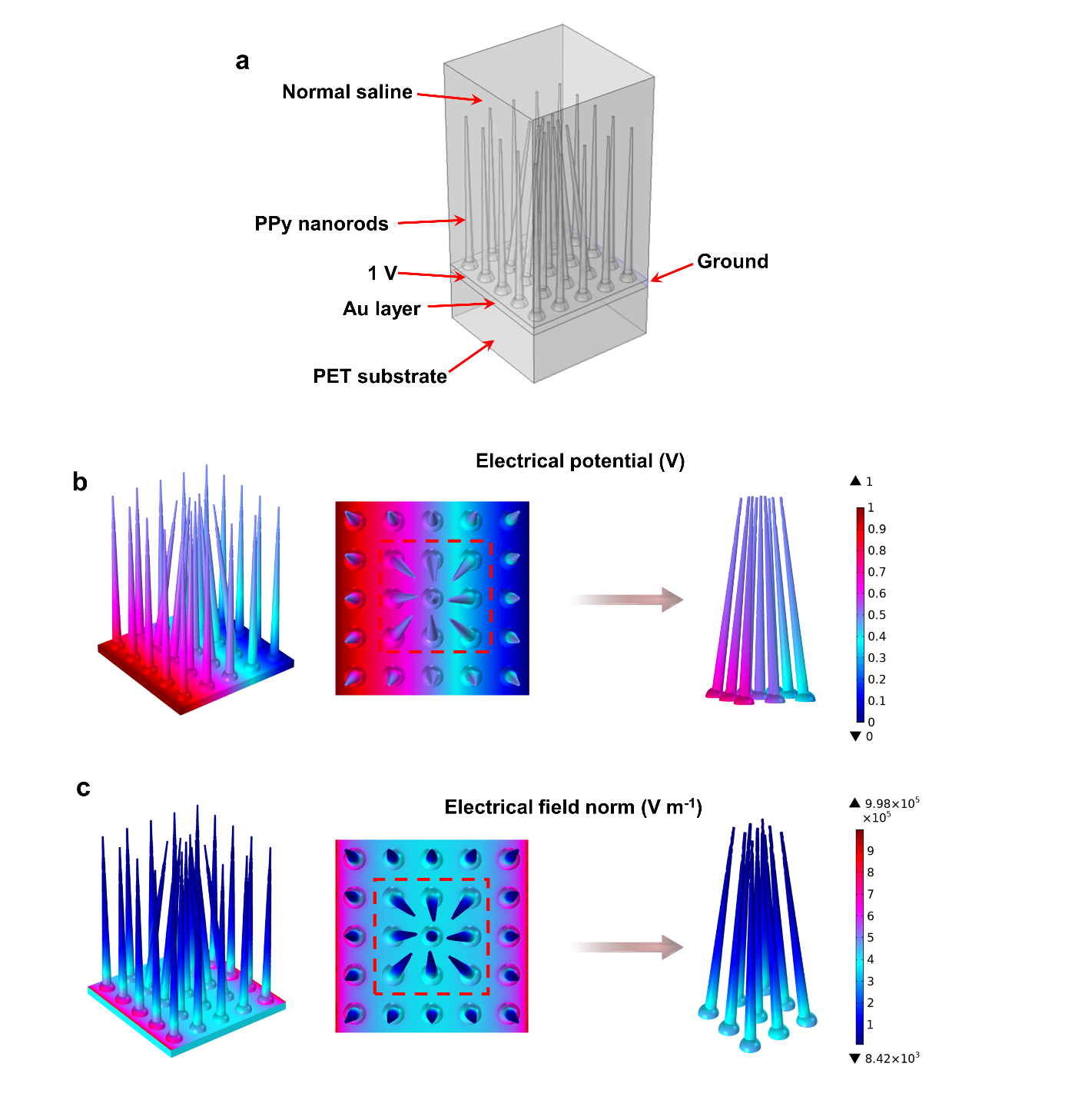
**

**Figure S11.** Simulation of the PNR mode: (a) Model, (b) Electrical potential distribution, and (c) Electric field distribution around the Au film and nanorod surface.

**
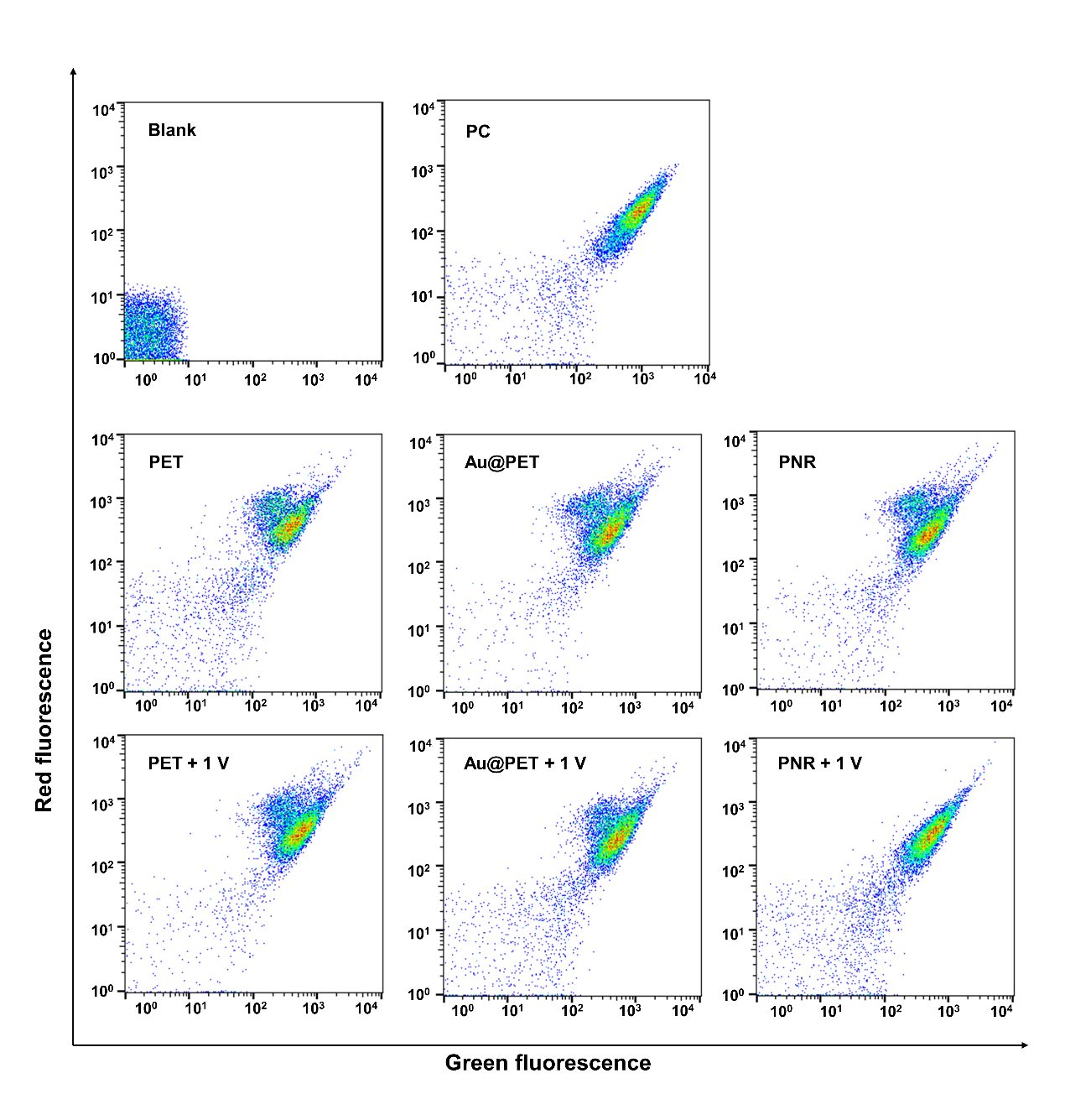
**

**Figure S12.** *E. coli* emitting red/green fluorescence after staining with the bacterial membrane potential kit.

**
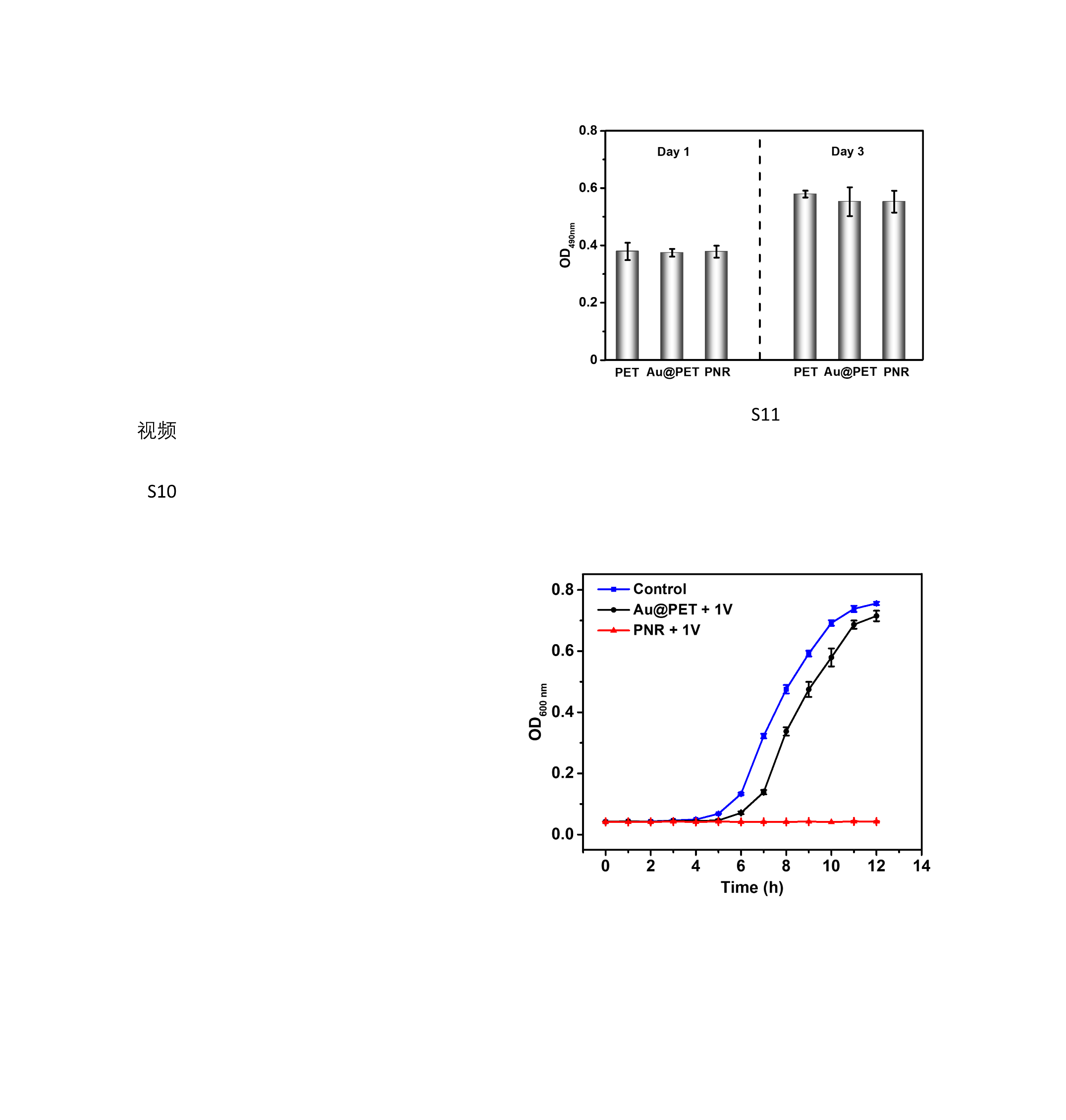
**

**Figure S13.** Growth curves of bacteria cultured on Au@PET and PNR after electrifying with solar power for 3 minutes.

**
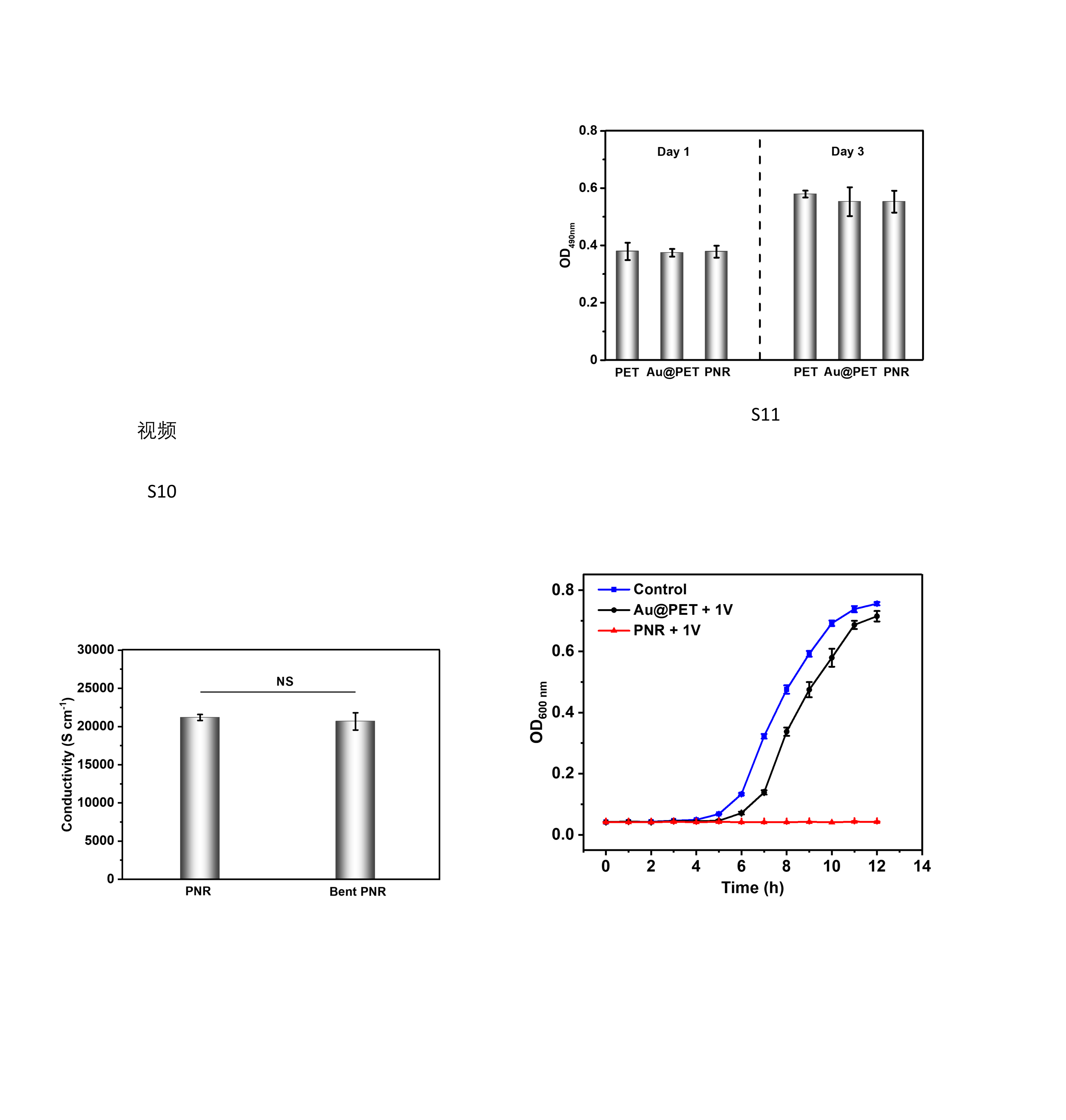
**

**Figure S14.** Conductivity changes of PNR under bending status and NS presents no significant difference.

**
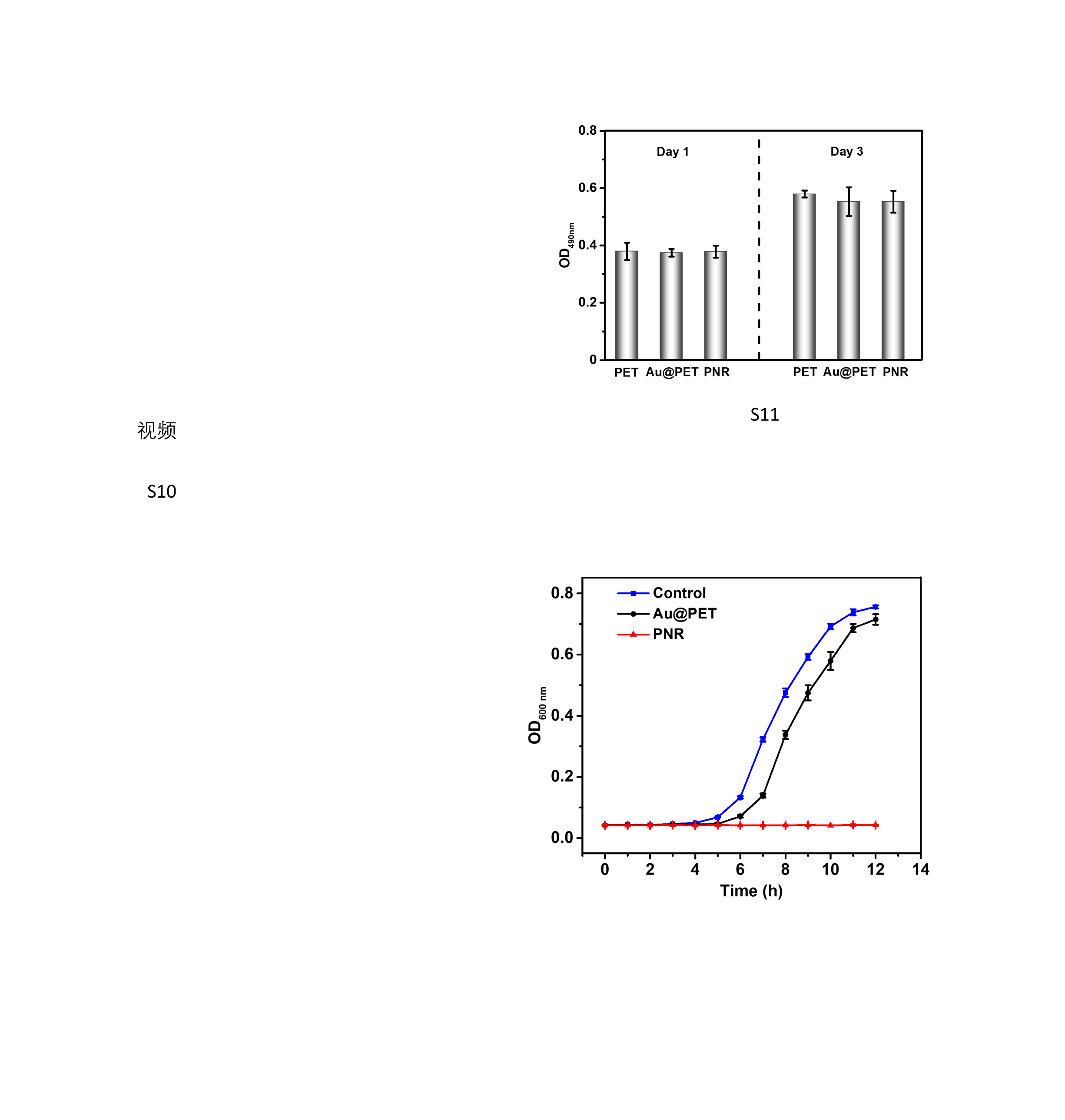
**

**Figure S15.** MTT assay of MRC-5 fibroblasts after culturing with different samples for 1 and 3 days.


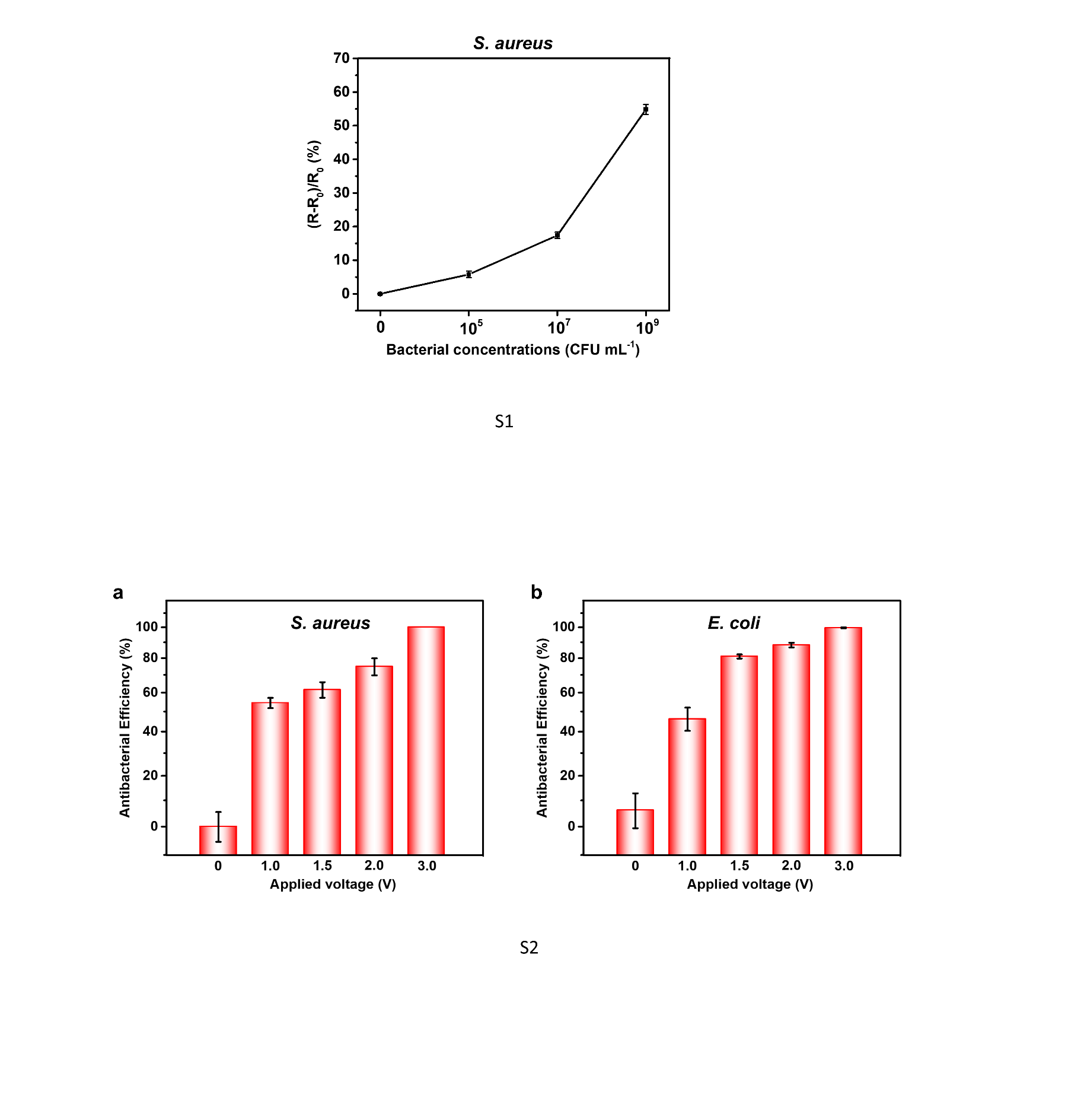


**Figure S16.** Antibacterial efficiency of Au-deposited PET films against *S. aureus* and *E. coli* at different applied voltages.

**Table S1.** Characteristic Raman peaks of PET and PNR:^[1-4]^

| Sample | | Raman shift (cm^-1^) | Assignment  Symmetry | Sample | Raman shift (cm^-1^) | | Assignment  Symmetry |
| --- | --- | --- | --- | --- | --- | --- | --- |
|  | 632 | C-C-C ring in-plane bending |  | 931 | Ring deformation | |  |
|  | 861 | C-C breathing |  | 1047 | Symmetrical C-H in-plane bending and N-H in-plane deformation | |  |
|  | 1000 | C-C stretching |  | 1331 | Antisymmetric in-ring  C-N stretching | |  |
| PET | 1097 | C-O stretching | PNR | 1420 | C-C and C-N stretching | |  |
|  | 1125 | C-H in-plane bending vibrations |  | 1595 | An overlap of C=C in-ring and C-C inter-ring stretching | |  |
|  | 1294 | Ring and C-O stretching |  |  |  | |  |
|  | 1618 | C=C ring stretching |  |  |  | |  |
|  | 1730 | C=O stretching |  |  |  | |  |

**References:**

[1] Y. Wu, Q. Ruan, C. Huang, Q. Liao, L. Liu, P. Liu, S. Mo, G. Wang, H. Wang, P. K. Chu, *Biomater. Adv.* **2022**, 112701, <https://doi.org/10.1016/j.msec.2022.112701>.

[2] S. C. Xin, N. Yang, F. Gao, J. Zhao, L. Li, C. Teng, *Appl. Surf. Sci.* **2017**, *414*, 218, <https://doi.org/10.1016/j.apsusc.2017.04.109>.

[3] Z. W. Zuo, K. Zhu, C. Gu, Y. B. Wen, G. L. Cui, J. Qu, *Appl. Surf. Sci.* **2016**, *379*, 66, <https://doi.org/10.1016/j.apsusc.2016.04.022>.

[4] T. Lippert, F. Zimmermann, A. Wokaun, *Appl. Spectrosc.* **1993**, *47* (11), 1931, https://doi.org/10.1366/0003702934065911.
